# Genetic associations with radiological damage in rheumatoid arthritis: Meta-analysis of seven genome-wide association studies of 2,775 cases

**DOI:** 10.1371/journal.pone.0223246

**Published:** 2019-10-09

**Authors:** Matthew Traylor, Rachel Knevel, Jing Cui, John Taylor, Westra Harm-Jan, Philip G. Conaghan, Andrew P. Cope, Charles Curtis, Paul Emery, Stephen Newhouse, Hamel Patel, Sophia Steer, Peter Gregersen, Nancy A. Shadick, Michael E. Weinblatt, Annette Van Der Helm-van Mil, Jennifer H. Barrett, Ann W. Morgan, Cathryn M. Lewis, Ian C. Scott

**Affiliations:** 1 Department of Clinical Neurosciences, Stroke Research Group, University of Cambridge, Cambridge, United Kingdom; 2 Department of Medical and Molecular Genetics, King’s College London, London, United Kingdom; 3 Brigham and Women’s Hospital, Division of Genetics, Raychaudhuri Lab, Boston, MA, United States of America; 4 Broad institute, Cambridge, MA, United States of America; 5 Department of Rheumatology C1-R, Leiden University Medical Center, Albinusdreef, Leiden, the Netherlands; 6 Division of Rheumatology Immunology and Allergy Brigham & Women's Hospital Harvard Medical School Boston, MA, United States of America; 7 Leeds Institute of Cancer & Pathology, Worsley Building Level 11 (LIDA), Clarendon Way, Leeds, United Kingdom; 8 Leeds Institute of Rheumatic and Musculoskeletal Medicine, University of Leeds, Leeds, United Kingdom; 9 NIHR Leeds Biomedical Research Centre, Leeds Teaching Hospitals NHS Trust, Leeds, United Kingdom; 10 Academic Department of Rheumatology, Centre for Molecular and Cellular Biology of Inflammation, King's College London, London, United Kingdom; 11 NIHR Biomedical Research Centre at South London and Maudsley NHS Foundation Trust and King’s College London, London, United Kingdom; 12 SGDP Centre, Institute of Psychiatry, Psychology & Neuroscience, King’s College London, London, United Kingdom; 13 Department of Biostatistics and Health Informatics, Institute of Psychiatry, Psychology and Neuroscience, King’s College London, London, United Kingdom; 14 Farr Institute of Health Informatics Research, UCL Institute of Health Informatics, University College London, London, United Kingdom; 15 Department of Rheumatology, King’s College Hospital, Denmark Hill, London, United Kingdom; 16 The Feinstein Institute for Medical Research, Northwell Health, Manhasset, New York, United States of America; 17 School of Medicine, University of Leeds, Leeds, United Kingdom; 18 Primary Care Centre Versus Arthritis, Research Institute for Primary Care and Health Sciences, Primary Care Sciences, Keele University, Keele, United Kingdom; 19 Haywood Academic Rheumatology Centre, Haywood Hospital, Midlands Partnership NHS Foundation Trust, High Lane, Burslem, Staffordshire, United Kingdom; Keio University, JAPAN

## Abstract

**Background:**

Previous studies of radiological damage in rheumatoid arthritis (RA) have used candidate-gene approaches, or evaluated single genome-wide association studies (GWAS). We undertook the first meta-analysis of GWAS of RA radiological damage to: (1) identify novel genetic loci for this trait; and (2) test previously validated variants.

**Methods:**

Seven GWAS (2,775 RA cases, of a range of ancestries) were combined in a meta-analysis. Radiological damage was assessed using modified Larsen scores, Sharp van Der Heijde scores, and erosive status. Single nucleotide polymophsim (SNP) associations with radiological damage were tested at a single time-point using regression models. Primary analyses included age and disease duration as covariates. Secondary analyses also included rheumatoid factor (RF). Meta-analyses were undertaken in trans-ethnic and European-only cases.

**Results:**

In the trans-ethnic primary meta-analysis, one SNP (rs112112734) in close proximity to *HLA-DRB1*, and strong linkage disequilibrium with the shared-epitope, attained genome-wide significance (*P* = 4.2x10^-8^). In the secondary analysis (adjusting for RF) the association was less significant (*P* = 1.7x10^-6^). In both trans-ethnic primary and secondary meta-analyses 14 regions contained SNPs with associations reaching *P*<5x10^-6^; in the European primary and secondary analyses 13 and 10 regions contained SNPs reaching *P*<5x10^-6^, respectively. Of the previously validated SNPs for radiological progression, only rs660895 (tagging HLA-DRB1*04:*01*) attained significance (*P* = 1.6x10^-5^) and had a consistent direction of effect across GWAS.

**Conclusions:**

Our meta-analysis confirms the known association between the *HLA-DRB1* shared epitope and RA radiological damage. The lack of replication of previously validated non-HLA markers highlights a requirement for further research to deliver clinically-useful prognostic genetic markers.

## Background

Rheumatoid arthritis (RA) is a heterogeneous disease, exhibiting a variable course between patients. Prospectively identifying patients likely to develop severe phenotypes could allow early intensive therapy to be focussed on poor prognosis cases. This approach should optimise clinical and cost-effectiveness, but requires accurate prognostic markers.

Radiological damage is one measure of RA severity. It is moderately heritable (heritability from common variants estimated at 45%-58%) suggesting genetic markers could represent useful prognostic biomarkers [1]. The strongest genetic association with RA radiological progression is the shared epitope (SE), which represents consensus amino acid sequences (QRRAA, RRRAA and QKRAA) spanning positions 70–74 in the HLA-DRβ1 molecule, encoded by various *HLA-DRB1* SE alleles [2]. The SE has been demonstrated to associate with greater radiological damage in a broad range of RA populations [3,4], although as it associates with the presence of rheumatoid factor (RF) and antibodies to citrullinated peptide antigens (ACPA) [5], both of which independently associate with radiological damage [6], its impact may be mediated by autoantibody status. Van Steenbergen *et al* previously identified a further 16 “validated” non-HLA variants (in 11 genes) for RA radiological progression [7]. In the Leiden Early Arthritis Cohort (EAC) these variants, in combination with the SE, explained up to 18% of the variance in radiological progression, although as this cohort was used to identify many of the variants, this finding requires replication.

The gold standard to identify genetic associations with a phenotype or trait is to perform a meta-analysis of all available genome-wide association studies (GWAS). To date, this has not been performed for RA radiological damage. Five individual GWAS have identified four non-HLA variants − rs7607479 in *SPAG16*, rs451066 in *RAD51L1-ZFP36L1*, rs11908352 in *SLC12A5*, and rs2833522 in *HUNK/SCAF4* –associating with RA radiological damage that passed multiple testing correction thresholds, and replicated externally [8–11]. These studies were limited by modest sample sizes, with the largest containing 646 patients. Combining GWAS in a meta-analysis should increase statistical power to detect novel loci.

To this end, we have carried out the largest GWAS of RA radiological damage, by peforming and then combining seven independent GWAS (totalling 2,775 patients). We aimed to identify novel genetic loci for radiological damage. We also tested previously validated single nucleotide polymorphisms (SNPs) for their association with radiological damage.

## Methods

### Patients

GWAS were undertaken in: (1) Combination Anti-Rheumatic Drugs in Early RA (CARDERA) Genetics Cohort; (2) Yorkshire Early Arthritis Registry (YEAR); (3) Brigham and Women’s Hospital RA Sequential Study (BRASS); (4) Leiden Early Arthritis Clinic (EAC); (5) North American RA Consortium (NARAC); (6) GENetics of RA in individuals of African ancestry (GENRA) study; (7) South London RA Study (SLRAS). These cohorts have been described in detail previously [12–17]. An overview is provided in Table 1 (with further details in S1 Table).

### Radiological measures

Hand and feet X-rays were scored using either the Scott modification of the Larsen method (scoring 0–200 for joint space narrowing and erosive damage [18]), Sharp van Der Heidje Scores (SvHS; scoring 0–448 for joint space narrowing and erosive damage [19]) or erosions (present/absent). Larsen and SvHS are highly correlated; both evaluate erosions [20].

### Genotyping, quality control and imputation

CARDERA was genotyped on the Immunochip, YEAR on the ImmunoChip and HumanOmniExpressExome Beadchip, BRASS on the Affymetrix 6.0, Leiden EAC on the Illumina iScan, GENRA on the Illumina MEGA array, NARAC on the Illumina Beadchip (HumanHap 550k), and SLRAS on the Illumina HumanOmniExpress. Prior to imputation all cohorts excluded SNPs with high levels of missingness, low minor allele frequency (MAF), and deviations from Hardy-weinberg equilibrium (HWE). Individuals were removed that did not segregate with reference populations, had high-levels of missingness, or whose phenotypic sex mismatched with sex inferred from genotype data. Genotype phasing and imputation were performed using SHAPEIT and IMPUTE2, respectively. Data were imputed to the 1,000 Genomes Phase 3 Panel, including all samples regardless of ancestry. Post-imputation markers with INFO scores <0.7 or MAF <0.05 were removed (the latter was undertaken as we had limited power to detect associations with low frequency variants). Genotypes were analysed as expected allele “dosages” in all analyses. Genotypes were converted to be reported on the forward strand. The number of variants available post-QC comprised: CARDERA: 3,181,676; YEAR: 6,209,766; Leiden EAC: 674,614; BRASS: 6,059,126; GENRA: 5,662,513; NARAC: 5,373,610; SLRAS: 5,715,821.”

### Statistical analysis

#### X-ray time-point

As RA X-ray progression can be non-linear [21], and several cohorts had cross-sectional X-ray scores, we tested SNP associations with radiological damage at a single time-point. In the repeated measures early RA cohorts (CARDERA, YEAR, and Leiden EAC) associations with end-point X-ray scores were tested (when radiological damage was greatest). In the repeated measures established RA cohort (BRASS) associations with baseline X-ray scores were tested (when sample size was greatest).

#### Statistical models

Log-transformed Larsen/SvHS, or binary erosive status were used as response variables. Outcomes were regressed on imputed genotype dosages assuming an additive genetic model using linear (in CARDERA, Leiden EAC, BRASS, SLRAS, and NARAC), negative binomial (in YEAR, due to over-dispersion) or logistic (in GENRA) regression models.

The same modelling covariates were included to provide consistency. Sex, age, disease duration, rheumatoid factor (RF) and (where available) treatment were tested for associations with X-ray scores in each cohort to determine the best predictors across studies. Only age and disease duration were associated with radiological scores in every cohort, and these were included as covariates. Ancestry-informative principal components (PCs) were included to account for population stratification. These were calculated using EIGENSTRAT. Different numbers were used in each study, with a minimum of 2 used [22].

Two analyses were undertaken: a primary analysis using these covariates, and a secondary analysis, including RF as an additional covariate to account for genetic variants exerting their effects through RF formation (which associates with both the SE and X-ray damage).

#### Meta-analysis

SNP association results from GWAS were combined using a Z-score weighted approach in METAL [23]. METAL performs a meta-analysis using information on *P*-values, effect directions, and the number of cases evaluated at each SNP. This method was used because other meta-analysis methods, such as the inverse variance-weighted average method [24], require effect size data at each marker, and as our various GWAS utlised different statistical models and X-ray scoring systems, their beta-values were non-comparable.

We assessed QQ-plots and genomic inflation factors to confirm that genome-wide statistics had the expected distributions. Genomic control correction was used to adjust for any residual inflation of test statistics. In the overall meta-analysis of all SNPs, we only analysed SNPs that had been genotyped or imputed in at least half of all individuals in the total sample.

Two meta-analyses were performed: (1) a trans-ethnic meta-analysis (maximising sample size); (2) a European ancestry meta-analysis (reducing heterogeneity across populations).

#### Associations with previously validated SNPs

Van Steenbergen *et al* reported 17 validated variants for radiological progression. These comprised variants in the following 12 loci: *SPAG16*, *IL*-15, *C5orf30*, *HLA-DRB1*, *OPG*, *DKK-1*, *IL2RA*, *RAD51L1/ZFP36L1*, *GRZB*, *IL-4R*, *MMP-9*, and *CD40*. They defined “validated” as being a SNP studied in several cohorts, with the association independently replicated or found significant in a meta-analysis of all published data [7]. We tested their association with radiological damage in our meta-analysis. We included all available SNPs, regardless of if they were present in less than half of all individuals. We used the HLA-DRB1*04:*01* tagging SNP (rs660895) identified in the Eyre *et al* RA susceptibility meta-analysis [25] to represent the SE, as this is the commonest SE-encoding allele in RA cases [2].

#### Significance thresholds

For the testing of previously validated SNPs we used a Bonferroni corrected *P*-value of 0.004167 (12 loci tested). For the genome-wide analyses we used the standard *P*-value threshold of <5x10^-8^ for genome-wide significance, and <5x10^-6^ for “suggestive” significance. In the genome-wide trans-ethnic analysis, based on sampling from a Chi-squared distribution with $NCP=\frac{Nq^{2}}{1-q^{2}}$, where q is trait variance and N is sample size, we had 80% power to detect a variant explaining 1.4% of the variance of X-ray damage at *P*<5x10^-8^ [26]. We made assumptions to enable us to perform a power calculation for this heterogeneous dataset. As most datasets used the quantitative Larsen scale, we simulated chi-squared statistics with an NCP given by N*qtl_variance/(1-qtl_variance) and calculated power based on the number of observations reaching genome-wide significance, using the principles reported by Yang et al [26]. We assumed we had 80% study power for qtl_variance values which produced genome-wide significant values for 80% of simulations.

### Ethics approval and consent to participate

Ethical approval was granted for each of the genetic studies as follows: CARDERA genetics cohort was approved by the National Research Ethics Service Committee East of England—Essex (reference: 11/EE/0544); YEAR by the Multi-Centre Research Ethics Committee (MREC) (reference 99/3/48); Leiden EAC by the Medical Ethics Committee Leiden University Medical Center; BRASS by the Institutional Review Board of Brigham and Women’s Hospital, Partners Health Care, Boston, MA; GENRA by the National Research Ethics Service Committee London—Dulwich (reference: 11/LO/1244); NARAC by the Via Christi Institutional Review Board and North Shore-LIJ Health System Institutional Review Board; SLRAS by the Guy's Hospital Local Research Committee (reference 99/11/06) and Lewisham Hospital Local Research Committee (reference 01/05/02). Informed consent was provided by all participating patients.

### Availability of data and materials

Individual-level genotype and phenotype data from each of the separate GWAS are not publically available, as the centres from which patients were recruited are reported in the primary publications outlining the individual genetic datasets, leading to the potential for patient confidentiality to be affected. Summary statistics will be made available through the NHGRI-EBI GWAS Catalog (https://www.ebi.ac.uk/gwas/downloads/summary-statistics). Further data requests can be made to the following non-author contacts: Ms Isabel Sinha (isabel.sinha@kcl.ac.uk) for CARDERA, GENRA, and SLRAS; Dr James Robinson (J.I.Robinson@leeds.ac.uk) for YEAR; Vivi Feathers (vfeathers@bwh.harvard.edu) for BRASS; Dr Annette Lee (Anlee@northwell.edu) for NARAC; and Dr E Niemantsverdriet (E.Niemantsverdriet@lumc.nl) for the Leiden EAC.

## Results

### Cohort characteristics

Cohort characteristics are presented in Table 1. The majority of cases were female (67.2–80.8%), and seropositive (57.4–100% RF-positive; 52.8–100% ACPA-positive). The mean age in all cohorts was between 54.5 and 60.9 years, except NARAC, which had a mean age of 40.8 years. As expected, the cohorts containing established RA patients had higher rates of radiological damage than those evaluating early RA patients. The total number of patients included in the trans-ethnic and European meta-analyses comprised 2,775 and 2,527 cases, respectively. The total number of SNPs assessed in the trans-ethnic meta-analysis was 4,802,696, and in the European meta-analysis was 2,723,488.

**Table 1:** Cohort characteristics.

Characteristic	CARDERA (n = 505) [12]	YEAR (n = 403) [13]	BRASS (n = 422) [14]	SLRAS (n = 284) [17]	GENRA (n = 196) [16]	Leiden EAC (n = 595) [13]	NARAC (n = 370) [15]
Ancestry	European	European	European	Mixed	African	European	European
Recruitment Site	England	England	North America	England	England	Netherlands	North America
Female, n (%)	345 (68.7)	278 (69.0)	341 (80.8)	227 (80.0)	164 (83.7)	400 (67.2)	272 (73.5)
Mean Age	54.5 (12.6)	60.3 (13.6)	58.9 (13.7)	60.9 (12.8)	56.2 (14.8)	57.1 (15.6)	40.8 (11.9)
Mean Disease Duration in Yrs (SD)	2.28 (0.41)	2.21 (1.17)	17.0 (12.7)	15.5 (11.6)	9.3 (10.2)	4.9 (2.2)	14.1 (10.6)
RF-Positive, n (%)	340 (67.3)	279 (69.2)	422 (100)	210 (75.5)	153 (78.1)	340 (57.4)	Unavailable
ACPA-Positive, n (%)	342 (67.7)	195 (65.9)	422 (100)	Unavailable	152 (77.6)	308 (52.8)	370 (100)
X-ray Scoring	Larsen	SvHS	SvHS	Larsen	Erosions	SvHS	SvHS^a^
Follow-Up Duration	2 years	2 years	9 years	Cross-sectional	Cross-sectional	7 years	Cross-sectional
Median X-ray Score (IQR)^b^	13 (3–25)	0 (0–5)	25 (5–64)	47 (25–77)	Unavailable	18 (7–37)	21 (6–56)
Erosive, n (%)	Unavailable	100 (24.8)	Unavailable	Unavailable	78 (39.8)	570 (95.8)	319 (86.0)

a = in NARAC SvHS were available for hand X-rays only

b = X-ray score at 2 years of follow-up in CARDERA/YEAR, 6 years of follow-up in Leiden EAC, baseline in BRASS, and at a single time-point in SLRAS/GENRA/NARAC; disease duration is at the time of the X-ray score tested for its association with genetic variants.

### Trans-ethnic meta-analysis

**Fig 1:**
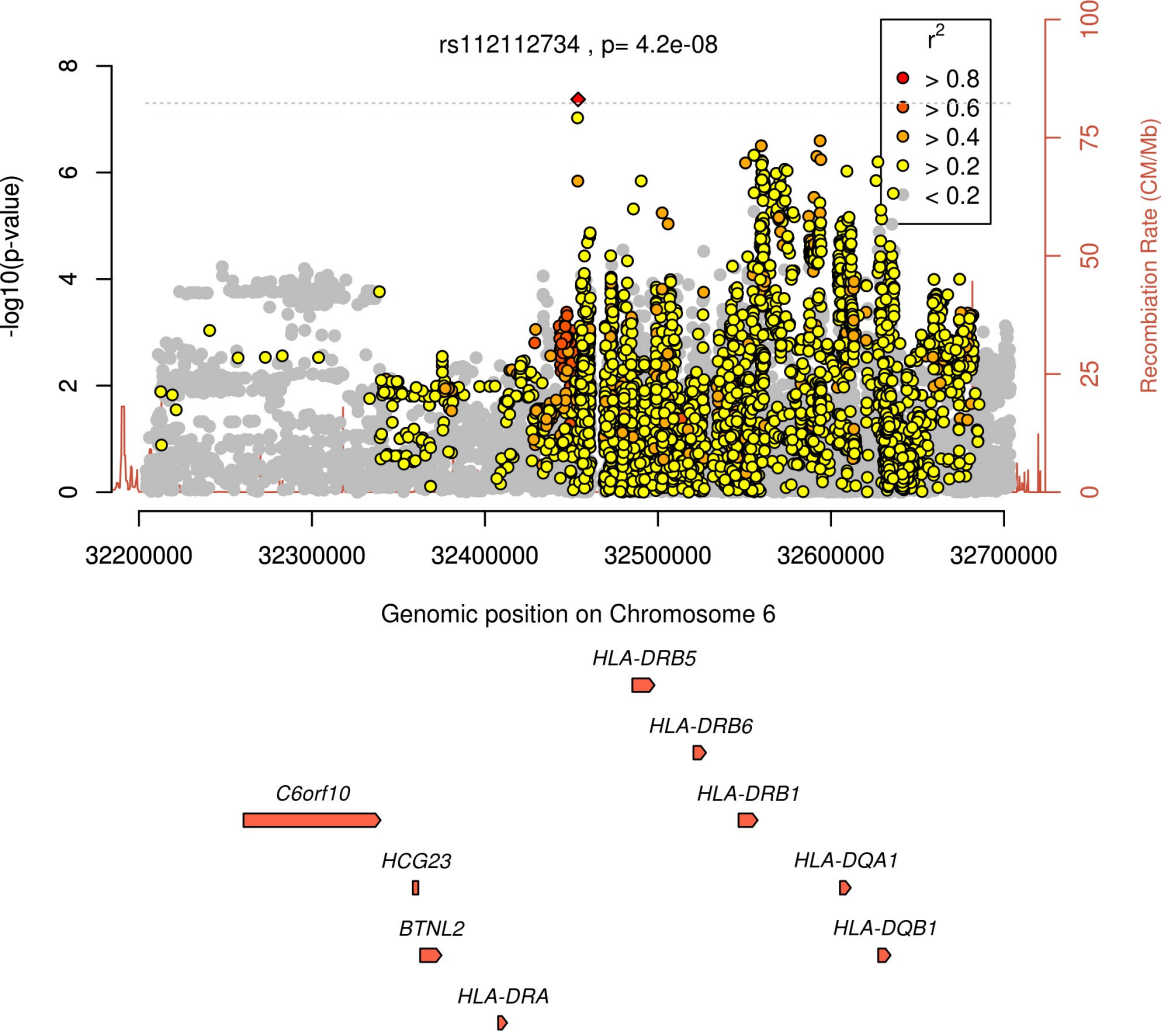
Regional association plot for region surrounding rs112112734 showing evidence of significance at *P*<5x10^-8^ in the primary trans-ethnic meta-analysis.

In the primary analysis (age and disease duration as covariates), one SNP (rs112112734, on chromosome 6, in close proximity to *HLA-DRB5*, *HLA-DRB6* and *HLA-DRB1*) attained genome-wide significance (*P* = 4.2x10^-8^, regional association plot in Fig 1). The same direction of effect was observed in the 6 GWAS in which this marker was available (it was absent in the Leiden EAC). This SNP is in strong linkage disequilibrium (LD) with the *HLA-DRB1* SE (R^2^ = 0.93 with the lead *HLA-DRB1* SNP, rs9268839, from the Okada *et al* RA susceptibility meta-analysis [27]). Fourteen regions contained SNPs with associations reaching *P*<5x10^-6^ (S2 Table).

**Fig 2:**
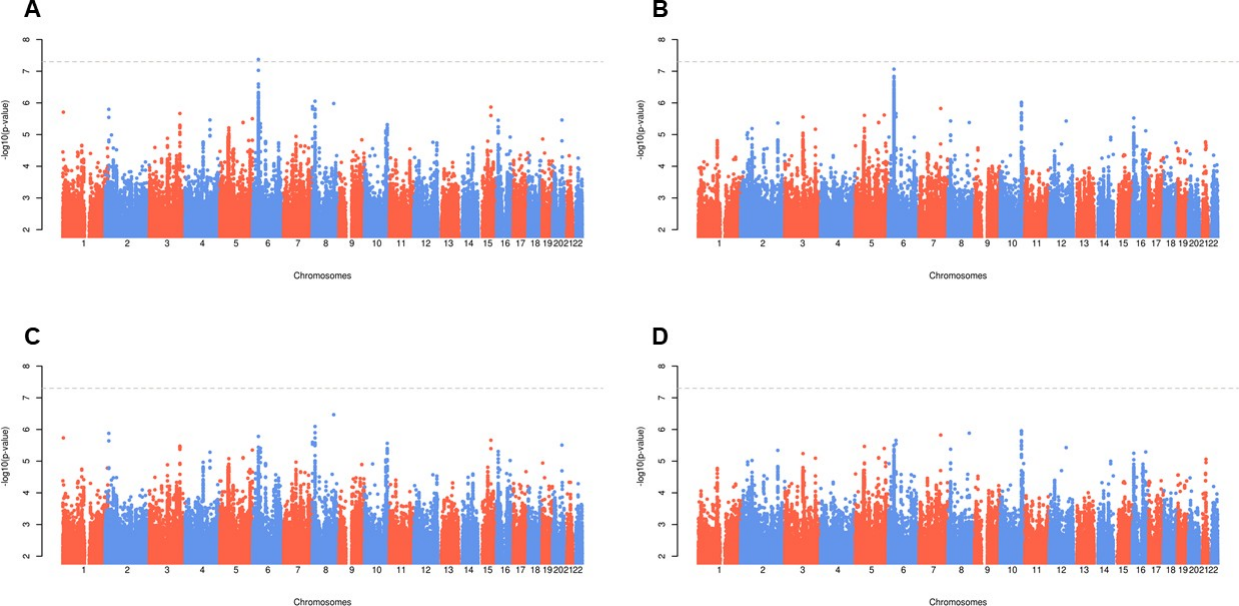
Manhattan plots for radiological damage. Panel A = trans-ethnic primary analysis; Panel B = European primary analysis; Panel C = trans-ethnic secondary analysis; Panel D = European secondary analysis; primary analysis includes age, disease duration and genotypes as explanatory variables; secondary analysis also includes RF as an explanatory variable.

In the secondary analysis (age, disease duration and RF as covariates), no SNPs attained genome-wide significance. Fourteen regions contained SNPs with associations reaching *P*<5x10^-6^, one of which was rs112112734 (*P* = 1.7x10^-6^) (S3 Table). Plots of–log_10_(*P*-value) by genomic position for the trans-ethnic and European analyses are provided in Fig 2, and QQ plots in S1 Fig.

### European meta-analysis

In the primary analysis, thirteen regions contained SNPs reaching *P*<5x10^-6^ (S2 Table), with the most significant SNP being rs112112734 (*P* = 8.6x10^-8^).

In the secondary analysis, ten regions contained SNPs with associations reaching *P*<5x10^-6^ (S3 Table). rs112112734 was not amongst these.

### Previously validated SNPs

Two SNPs associated with radiological damage in our European meta-analysis (Table 2). These comprised: (1) rs660895 (tagging HLA-DRB1*04:*01*; *P* = 1.6x10^-5^) which had the same direction of effect across all 6 GWAS (the G allele, indicating an *04:01 copy, associating with increased damage), and (2) rs7607479 (in *SPAG16*), which was available in four GWAS, two of which had increased and two reduced radiological damage associated with the T allele. In the trans-ethnic meta-analysis, only rs660895 was significant (*P* = 7.0x10^-5^) with the same direction of effect observed in all but one GWAS. Only 2 SNPs in the trans-ethnic meta-analysis, and 4 SNPs in the European meta-analysis had a shared direction of effect across all available GWAS.

**Table 2:** Previously validated genetic variants for radiological progression reported by Van Steenbergen et al [7], and their association with radiological damage.

					Trans-ethnic			European	
Variant	*Gene*	Chr	A1/A2	A1 Freq.	Cohort Direction	*P*	A1 Freq.	Cohort Direction	*P*
rs7607479	*SPAG16*	2	T/C	0.53	-+?-?++	4.4x10^-3^	0.55	-+?-?+	4.0x10^-3^
rs4371699	*IL-15*	4	A/C	0.33	++?+?++	0.0869	0.38	++?+?+	0.0445
rs6821171	*IL-15*	4	T/G	0.66	+--+?++	0.7913	0.65	+--+?+	0.5901
rs7665842	*IL-15*	4	T/C	0.65	-++-?-+	0.6102	0.63	-++-?-	0.4719
rs7667746	*IL-15*	4	T/C	0.60	-+?-?-+	0.3014	0.55	-+?-?-	0.1528
rs26232	*C5orf30*	5	T/C	0.45	--+--++	0.5763	0.46	--+--+	0.4193
rs660895	HLA-DRB1*04:*01*	6	A/G	0.56	--+--	7.0x10^-5^	0.54	------	1.6x10^-5^
rs1485305	*OPG*	8	A/T	0.50	+++-?+-	0.7832	0.53	+++-?+	0.5594
rs1528873	*DKK-1*	10	A/C	0.55	++?-?--	0.4979	0.53	++?-?-	0.6317
rs1896367	*DKK-1*	10	T/C	0.44	++?+?+?	0.1337	0.44	++?+?+	0.1304
rs1896368	*DKK-1*	10	T/C	0.47	++?+?+-	0.2077	0.50	++?+?+	0.1749
rs2104286	*IL2RA*	10	T/C	0.56	-+-++-?	0.8596	0.55	-+-++-	0.9270
rs451066	*RAD51L1*, *ZFP36L1*	14	A/G	0.41	-+-++-+	0.6024	0.44	-+-++-	0.6415
rs8192916	*GRZB*	14	A/G	0.43	+-?+??-	0.7444	0.39	+-?+??	0.7408
rs1119132	*IL-4R*	16	A/G	0.38	--+-+++	0.1923	0.41	--++++	0.1912
rs11908352	*MMP-9*	20	A/C	0.41	++--+-+	0.2055	0.43	++--+-	0.2169
rs4810485	*CD40*	20	T/G	0.41	+--+-+-	0.4666	0.44	+--+-+	0.4924

Cohort direction indicates the direction of the SNP A1 allele effect on X-ray damage across the cohorts (+ means it is associated with increased damage,—means it is associated with decreased damage, and ? means the SNP was unavailable in the cohort). Cohort order comprises YEAR, BRASS, CARDERA, SLRAS, Leiden, NARAC, GENRA in the trans-ethnic analysis and YEAR, BRASS, CARDERA, SLRAS, Leiden, and NARAC in the European analysis); results in the table are from the primary analysis (including age and gender as covariates). Note that (a) Stouffer’s meta-analysis method does not permit effect sizes to be calculated, and (b) the effect direction for the SLRAS cohort differs at two markers (rs660895 and rs1119132) because it is a mixed-ethnicity cohort and different individuals were included in the trans-ethnic and European analyses in this GWAS.

## Discussion

Our study has three key findings. Firstly, it confirms the known association between this trait and the HLA-region, with one SNP in this region (rs112112734, in high LD with the *HLA-DRB1* SE) attaining genome-wide significance. Secondly, it demonstrates the challenges in replicating genetic variants for traits across GWAS. Of the 17 previously validated SNPs, only the HLA-DRB1*04:*01* tagging SNP had a significant association and consistent direction of effect across GWAS. Thirdly, it highlights the difficulties in performing meta-analysis of GWAS of continuous disease outcomes like RA radiological damage, which involves combining summary statistics from highly heterogeneous patient cohorts.

The association between the SE and radiological damage in RA is well established [3]. As the SE associates with RF and ACPA, which themselves are linked with radiological damage, we undertook a secondary analysis including RF as a covariate (ACPA was not used, as ACPA status was unavailable in SLRAS, and two cohorts only included ACPA-positive cases). The association observed at chromosome 6 was less significant in this secondary analysis, and rs112112734 no longer reached genome-wide significance. This suggests the observed association between the HLA-region and X-ray damage in our meta-analysis may be, at least in part, mediated by autoantibody production. More recently, Viatte *et al* reported that amino acid polymorphisms at position 11 in HLA-DRβ1 also associate with erosions, independently of the shared epitope [28]; owing to an absence of imputed amino acid polymorphism data, and the fact that this amino acid polymorphism is not “tagged” by a single SNP, we were unable to evaluate this association within our meta-analysis. Future research should focus on fine mapping of the HLA locus to provide a more comprehensive understanding of the association between HLA alleles and X-ray damage.

Our failure to replicate associations for previously validated non-HLA markers is disappointing. There are several potential explanations for this. Firstly, the validated variants reported by Van Steenbergen *et al* were for radiological progression, and we tested their association with radiological damage (although only radiological “progressors” will accrue damage). Secondly, several of these variants were identified in ACPA-positive cases only, and our cohorts included cases with a range of frequencies of ACPA. Thirdly, not all SNPs were available in all cohorts, although only one SNP (rs8192916 in *GRZB*) had a sample size of <1,000 cases. Irrespective of the explanation, current data on seven GWAS strengthen the notion that these variants lack clinical utility at identifying severe phenotypes [7].

The strengths of our study are that it represents the largest analysis of genetic predictors of RA radiological damage, includes individuals from a range of ethnicities (increasing the generalisability of its results), and is the first meta-analysis of an RA prognostic trait. Its weaknesses are the marked heterogeneity of the included GWAS (which varied in their disease durations, X-ray scoring systems used, and statistical models), and its modest sample size, limiting our ability to detect variants of a small effect size. As our meta-analysis replicated the genetic locus with the largest effect size for radiological damage (the HLA region), we consider that our modest sample size is the most likely reason we did not identify non-HLA associations attaining genome-wide significance. An alternative explanation is that the trait of radiological damage has low heritability. Due to a modest sample size, and clinical heterogeneity, we could not determine heritability in our GWAS meta-analysis with confidence, although previous research has reported RA radiological damage to be moderately heritable (estimated heritability rate of 45–58%) [1].

## Conclusions

In conclusion, our meta-analysis replicates the association between the HLA-region and radiological damage in RA. It demonstrates the complexities of undertaking meta-analysis of GWAS of RA characteristics. For these to be more precisely defined, a collaborative international approach is required, standardising the collection of phenotypic data in genotyped patients, creating large homogeneous cohorts that are suitable for meta-analysis.

## Supporting information

S1 Table Genome-Wide association study details. (PDF)

S2 Table Lead SNPs from regions showing evidence of significance at *P*<5x10^-6^ in the primary trans-ethnic and European meta-analysis. (PDF)

S3 Table Lead SNPs from regions showing evidence of significance at *P*<5x10^-6^ in the secondary trans-ethnic and European meta-analysis. (PDF)

S1 Fig QQ plots for meta-analyses. A = primary trans-ethnic meta-analysis (inflation factor 1.00); B = primary European meta-analysis (inflation factor 1.01); C = secondary trans-ethnic meta-analysis (inflation factor 1.00); D = secondary European meta-analysis (inflation factor 1.00).
(TIF)
