# Supplementary material for: Genetic associations with radiological damage in rheumatoid arthritis: Meta-analysis of seven genome-wide association studies of 2,775 cases
Source: PLoS One. 2019 Oct 9;14(10):e0223246. doi: 10.1371/journal.pone.0223246 (PMC6785117; doi:10.1371/journal.pone.0223246)
Supplement: S1 Table — (PDF) [file pone.0223246.s001.pdf]

S1 Table. Genome-Wide Association Study Details

Cohort	Overview
CARDERA	The CARDERA genetics cohort comprises 524 early active RA patients of European ancestry previously enrolled to the CARDERA-1 and CARDERA-2 trials. The original CARDERA-1 and CARDERA-2 trials recruited 467 and 159 patients, respectively, from 42 English rheumatology units, and evaluated the impact of combination disease-modifying anti-rheumatic drug (DMARD) therapy and anakinra (an IL-1 receptor antagonist). X-rays were scored using the modified Larsen method every 6 months in CARDERA-1 and 12 months in CARDERA-2 for 2 years. The current meta-analysis includes the 505 patients with 2-year X-ray scores available. Genotyping was on the ImmunoChip. Ethical approval for the genetics cohort was granted by the National Research Ethics Service Committee East of England—Essex, reference: 11/EE/0544.
YEAR	YEAR recruited early RA patients from 14 centres in the UK Yorkshire region between 2000 and 2009. X-rays were scored for SvHS every 12 months for 2 years. The current meta-analysis includes 403 patients of European ancestry with genotype data available. Genotyping was on the ImmunoChip and HumanOmniExpressExome Beadchip. Ethical approval for this study was granted by the Multi-Centre Research Ethics Committee (MREC) (99/3/48).
Leiden EAC	Leiden EAC is a prospective observational study that recruits early arthritis patients with a maximum 1 year of symptoms. 646 consecutive RA patients with early RA that were included between 1993 and 2006 were genotyped and X-rays were scored for SvHS annually for 7 years. The current meta-analysis includes the 595 patients passing QC procedures. Genotyping was on the Illumina iScan. Ethical approval for this study was granted by the Medical ethics committee Leiden University Medical Center.
BRASS	BRASS is a prospective observational study established in 2003, recruiting over 1,000 patients with early and established RA attending rheumatology clinics at the Brigham and Women's Hospital (USA). X-rays were scored for SvHS at baseline, 2 and 5 years. The current analysis includes 422 ACPA-positive of European ancestry with genotype data available. Genotyping was on the Affymetrix 6.0. Ethical approval for this study was granted by the Institutional Review Board.
GENRA	GENRA is a cross-sectional study evaluating RA susceptibility factors in individuals of African ancestry. It recruited 212 early and established African ancestry RA patients from 4 centres in South London. X-rays were evaluated for the presence of erosions. The current analysis includes the 196 patients passing QC procedures. Genotyping was on the Multi-Ethnic-Genotyping-Array (MEGA). Ethical approval was granted by the National Research Ethics Service Committee London—Dulwich, reference: 11/LO/1244.
NARAC	NARAC is an observational cross-sectional study recruiting 512 multicase RA families, in whom at least one sibling had documented erosions on X-ray. Patients were recruited between 1985 and 2002. X-rays were scored for SvHS at a single time point. The current meta-analysis includes 370 patients (only one case from each family) passing QC procedures. Genotyping was on the Illumina Beadchip (HumanHap 550k). Ethical approval for this study was granted.
SLRAS	SLRAS is a cross-sectional study, which recruited 358 established RA patients of variable ancestry from 3 centres in South London. X-rays were scored using the modified Larsen method. The current meta-analysis includes the 284 patients with X-ray data available that passed QC procedures. Genotyping was on the HumanOmniExpress Beadchip. Ethical approval for this study was granted (Guy's Hospital LREC reference 99/11/06; Lewisham Hospital LREC reference 01/05/02).
