# Supplementary material for: Genetic associations with radiological damage in rheumatoid arthritis: Meta-analysis of seven genome-wide association studies of 2,775 cases
Source: PLoS One. 2019 Oct 9;14(10):e0223246. doi: 10.1371/journal.pone.0223246 (PMC6785117; doi:10.1371/journal.pone.0223246)
Supplement: S2 Table — (PDF) [file pone.0223246.s002.pdf]

S2 Table. Lead SNPs from Regions Showing Evidence of Significance at $P<5x10^{-6}$ in the Primary Trans-Ethnic and European Meta-Analysis

SNP	Chromosome	Base Position (hg19)	Gene	*P*
Trans-Ethnic				
rs10088748	8	14468525	*SGCZ*	8.80x10^{-07}
rs4129294	8	117475290	*LOC105375711*	1.00x10^{-06}
rs60603343	8	673082	*ERICH1*	1.30x10^{-06}
rs58999906	15	70155247	-	1.40x10^{-06}
rs72783289	2	20398295	-	1.60x$^{10-06}$
rs3765718	1	3603695	*TP73*	2.00x10^{-06}
rs7641666	3	169213690	*MECOM*	2.20x10^{-06}
rs34228803	5	179860167	-	3.20x10^{-06}
rs72984811	4	137222200	-	3.50x10^{-06}
rs73277715	20	50541548	-	3.50x10^{-06}
rs72763714	16	7933806	*LOC105371069*	3.50x10^{-06}
rs115275214	5	128738910	-	4.10x10^{-06}
rs2277125	6	43640099	*MRPS18A*	4.50x10^{-06}
rs2486037	10	125380508	-	4.80x10^{-06}
European				
rs112112734	6	32453853	-	8.60x10^{-08}
rs180558	10	117814519	-	9.60x10^{-07}
rs78619335	7	119037514	-	1.50x10^{-06}
rs2295948	6	43646401	*MRPS18A*	2.20x10^{-06}
rs4594895	5	159670626	-	2.40x10^{-06}
rs76166785	5	50260095	-	2.50x10^{-06}
rs7646803	3	100797038	-	2.80x10^{-06}
rs7186844	16	7931285	*LOC105371069*	3.00x10^{-06}
rs17249839	8	14433115	*SGCZ*	3.70x10^{-06}
rs11107128	12	94007289	*SOCS2*	3.70x10^{-06}
rs115275214	5	128738910	-	4.10x10^{-06}
rs4129294	8	117475290	*LOC105375711*	4.20x10^{-06}
rs147138549	2	204927705	-	4.30x10^{-06}
