# Supplementary figures and images for: Genetic associations with radiological damage in rheumatoid arthritis: Meta-analysis of seven genome-wide association studies of 2,775 cases
Source: PLoS One. 2019 Oct 9;14(10):e0223246. doi: 10.1371/journal.pone.0223246 (PMC6785117; doi:10.1371/journal.pone.0223246)

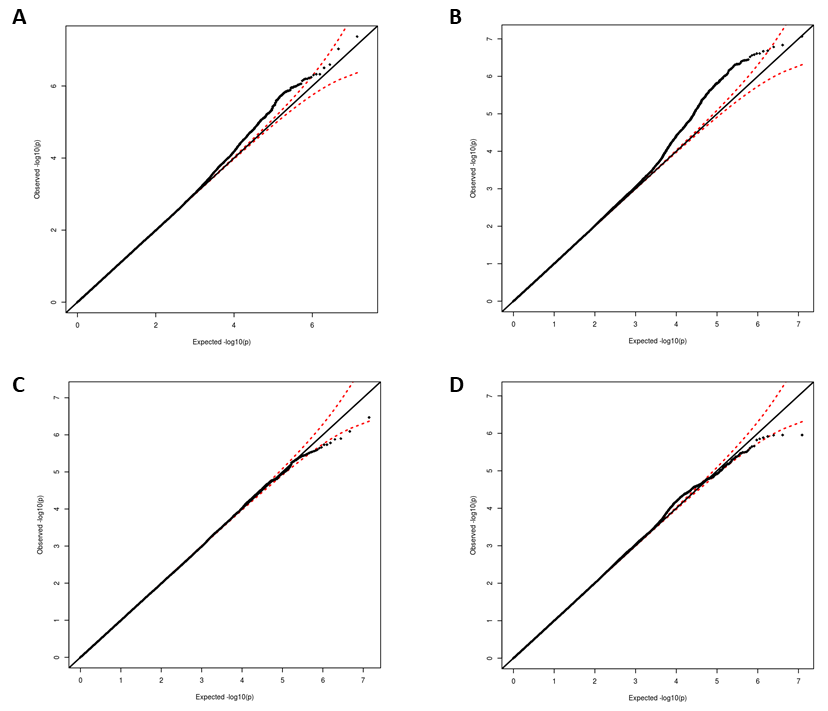

Supplement: S1 Fig — A = primary trans-ethnic meta-analysis (inflation factor 1.00); B = primary European meta-analysis (inflation factor 1.01); C = secondary trans-ethnic meta-analysis (inflation factor 1.00); D = secondary European meta-analysis (inflation factor 1.00). (TIF) [file pone.0223246.s004.tif]
